# Influences of First and Second Language Phonology on Spanish Children Learning to Read in English

**DOI:** 10.3389/fpsyg.2022.803518

**Published:** 2022-03-24

**Authors:** Carmen Hevia-Tuero, Sara Incera, Paz Suárez-Coalla

**Affiliations:** ^1^Laboratorio de Psicología del Lenguaje, Department of Psychology, University of Oviedo, Oviedo, Spain; ^2^Multilingual Laboratory, Department of Psychology, Eastern Kentucky University, Richmond, KY, United States

**Keywords:** orthography, phonology, bilingual reading, pseudohomophones, mouse-tracking

## Abstract

Children learning to read in two different orthographic systems are exposed to cross-linguistic interferences. We explored the effects of school (Monolingual, Bilingual) and grade (2nd, 4th, and 6th) on phonological activation during a visual word recognition task. Elementary school children from Spain completed a lexical decision task in English. The task included real words and pseudohomophones following Spanish or English phonological rules. Using the mouse-tracking paradigm, we analyzed errors, reaction times, and computer mouse movements. Children in the bilingual school performed better than children in the monolingual school. Children in higher grades performed better than children in lower grades. The interference effect of Spanish phonology was weak and became weaker in higher grades. Spanish children differentiate between first and second language grapheme-to-phoneme correspondences since early on in the educational process. In 6th grade, children from the bilingual school responded better to words and Spanish pseudohomophones, while children from the monolingual school were less distracted by the English pseudohomophones. Children in the bilingual school had stronger inhibition of Spanish (L1) phonology and stronger activation of English (L2) phonology. Instructional method plays an important role on the processing strategies Spanish children rely on when reading in English. School and grade influence the link between orthographic and phonological representations.

## Introduction

Learning to read is a key foundation for education, and much effort is invested in ensuring all children are able to read properly. Learning a second language is also important, as it allows worldwide communication and it improves professional development. Thus, how children learn to read in a second language is an important topic to investigate.

Speaking more than one language is an important skill highly valued within the European educational systems (Council of Europe, 2001). In Spain, studying a foreign language at school is compulsory for all children. English is by far the most popular, and the number of schools implementing Spanish–English bilingual programs is increasing. Many bilingual education programs are being developed, but English as a second language instructional methods vary across schools (Hélot and Cavalli, 2017). The consequences of this variety of educational approaches have not been fully investigated, but these different techniques could be influencing how children learn to read in a second language. For instance, it has been demonstrated that being more exposed to second language impacts positively on language learning (Farukh and Vulchanova, 2015). Thus, it is essential to determine the role of school and to ensure teachers know how to help students read in their second language. Despite the undeniable benefits of being exposed to a second language since early stages (Winsler et al., 1999; Larson-Hall, 2008; Olulade et al., 2016), children face the challenge of simultaneously learning to read and write in two different orthographies. The purpose of this study is to determine how Spanish children learn to read in English. In particular, we examine the effect of school and grade on word processing during second language reading.

When children learn to read in their native language, they learn a specific set of grapheme-to-phoneme correspondences. The goal is to connect a grapheme (the letter “a”) to its correspondent phoneme (the sound/ʌ/). For instance, in order to form the word cat, the letters c - a - t are processed and connected to the sound each is related to. In languages like English, it may also be necessary to learn correspondences between larger segments of writing, like syllables or rhymes, and their phonological representations. Regardless of the size of the processing units (as long as it is not the whole word) this serial rule-based procedure is known as sublexical decoding (Rau et al., 2014). A stage of sublexical decoding is included in some reading developmental models (Frith, 1985; Ehri, 2005). In later stages, readers transit from this sublexical to a lexical strategy, which improves fluency and efficiency. However, as Share (1995) states in the self-teaching hypothesis, this developmental transition can be different for each word and strategies may overlap. Every time a word is successfully decoded, children acquire specific orthographic information. The orthographic representation of the word will be formed through a self-teaching mechanism after repeated exposures. The coexistence of phonological and lexical processing continues along the reader’s life. This highlights the relevance of print-to-sound correspondence knowledge, which is specific to the orthography of each language (Goswami et al., 2001). Children learning to read are influenced by orthographic depth of their native language—the extent to which the orthography is a phonetic representation of speech (Katz and Feldman, 2017). This reliability of print-to-sound correspondences is based on the complexity and unpredictability of the orthography (Schmalz et al., 2015; De Simone et al., 2021). In more shallow orthographies (e.g., Spanish), each grapheme is associated with a single phoneme; there is a one-to-one correspondence with relatively few exceptions. However, in deeper orthographies (e.g., English) each grapheme can be associated with multiple phonemes. In these cases, the formation of strong orthographic representations and the transition from a sublexical to a lexical strategy will be more likely than in shallower orthographies.

Orthographic depth determines the main route (phonological or lexical) children rely on most during literacy acquisition (Ziegler and Goswami, 2005). For instance, children learning to read in a shallower orthography language like Spanish rely heavily on the phonological route and use more frequently grapheme–phoneme decoding strategies (Bhide, 2015). This facilitates code learning, allowing Spanish children to reach accuracy in reading sooner than their counterparts who learn to read in deeper orthography languages like English (Seymour et al., 2003). On the contrary, children learning to read in a deeper orthography like English rely more frequently on the lexical route (Defior and Serrano, 2005). Because not all graphemes correspond to a unique phoneme in English, children’s sublexical decoding is based on units bigger than graphemes (e.g., syllables). The orthographic context, as well as other sublexical elements like syllables or rhymes, must be taken into consideration in more deep orthographies. This makes decoding a more complex task for English than for Spanish readers, which results in children who are learning to read in English reaching reading accuracy about a year later than their Spanish counterparts.

In bilingual programs children are exposed to another language and must learn an additional set of grapheme-to-phoneme mappings. While English and Spanish share the same alphabet, the grapheme–phoneme equivalences are not the same. For instance, the sound /i/ is represented with i in Spanish and ee or ea in English. This sound is perceived in English as a long vowel, but vowel length is not a relevant aspect in Spanish (Fox et al., 1995). Furthermore, other phonemes may be perceived as two separate sounds in English but a single sound for Spanish speakers. For instance, the /ʤ/ in jeans (which is not contrastive with the /j/ in yellow) or the /i/ and /ɪ/, which are both perceived and represented as the same grapheme i. This substitution of the spelling of an English specific phoneme (like /i:/ or /ʌ/) for the spelling of the closest phoneme in Spanish (like/i/ or/a/) has been frequently reported (Cronnell, 1985; Zutell and Allen, 1988; Fashola et al., 1996; Sun-Alperin and Wang, 2008; Howard et al., 2012). In the case of cheese, for example, its transcription following Spanish rules would be chis. This lack of discrimination affects not only the vowel sound, but the final/z/ phoneme as well. This voice alveolar fricative does not exist in Spanish, and its closest phoneme is a voiceless alveolar fricative (/s/). Moreover, in Spanish the letter “z” represents the sound/θ/, which is normally spelled as “th” in English. These inconsistencies help illustrate the incongruences that Spanish children encounter when learning to read in English.

While understanding the orthography of each language is essential to learn how to read, the corresponding phonology also plays an important role in literacy acquisition. For instance, the triangle model (Seidenberg and McClelland, 1989; Harm and Seidenberg, 2004) suggests a cooperation between orthography and phonology to read words. Nevertheless, exposure to the phonology of both languages can lead to cross-linguistic interferences between first language (L1) and second language (L2; Akamatsu, 2003; Lemhöfer et al., 2008; Sun-Alperin and Wang, 2008; Deacon et al., 2009; Ota et al., 2010; Howard et al., 2012; Bhide, 2015). As posited by the language non-selective lexical access hypothesis (Dijkstra and van Heuven, 2002), lexical and sublexical information from both languages is coactivated during word reading. The strength of these influences depend on variables like exposure (Brysbaert et al., 2017), amount of use (Flege et al., 1997; Luk and Bialystok, 2013), proficiency in L1, L2, or both languages (Haigh and Jared, 2007; Van Hell and Tanner, 2012), age (Howard et al., 2012), and the specific orthography (Beauvillain, 1992; Bialystok et al., 2005a; Hamada and Koda, 2008; Lemhöfer et al., 2008; Sun-Alperin and Wang, 2008; Ota et al., 2010; Lallier and Carreiras, 2018) and phonology (Sun-Alperin and Wang, 2008; Ota et al., 2009, 2010) of the L1 and L2 languages. Confusion between decoding rules (e.g., reading an English word by applying Spanish phonological rules) is likely to influence bilingual readers when the languages differ in terms of orthographic depth (Goswami et al., 1998). Many authors suggest that early phonological activation of both L1 and L2 phonological codes overlap during reading (Jared and Szucs, 2002; Duyck, 2005; Jared et al., 2012). This overlap of the two languages happens even in skilled readers that rely on lexical strategies (Perfetti and Bell, 1991; Grainger et al., 2005; Braun et al., 2009).

The pseudohomophone effect provides consistent evidence of phonological activation during reading. Pseudohomophones are non-words that sound like real words (e.g., pseudohomophones of the real English word cheese would be /chease/ or /chis/). Pseudohomophones are orthographically different from words, but phonologically equivalent. In native speakers, pseudohomophones yield faster responses in naming, which reflects a facilitating effect of familiar pronunciations (McCann and Besner, 1987; Seidenberg et al., 1996; Goswami et al., 2001). In addition, pseudohomophones delay responses in lexical decision tasks; since they sound like real words it is more difficult to discard them efficiently (McCann et al., 1988; Seidenberg et al., 1996; Goswami et al., 2001; Pexman et al., 2001; Ziegler et al., 2001; Briesemeister et al., 2009).

The pseudohomophone effect can be explained by computational models of visual word recognition like the multiple read-out model (MROM-p; Jacobs et al., 1998) or the dual-route cascaded model (DRC; Coltheart et al., 2001). In the MROM-p, a stimulus is rejected as a non-word when a threshold is not reached within a certain amount of time. During the processing of a pseudohomophone, there is a mismatch in the activation of the phonological and orthographical nodes, which requires a readjustment that results in delays in the response. The DRC, implemented with the MROM-p, is based on the double-route model (Coltheart, 1978). According to this model, activation in early modules flows to later modules, which receives excitation or inhibition from feedback pathways. In this model, a pseudohomophone activates a lexical entry in the phonological lexicon that does not match with any input in the orthographical lexicon, producing an incongruity. Both models describe a conflict between the “real word” phonological information and the “non-word” orthographical information. Readers are able to resolve this conflict, but the time needed to do so results in delayed responses.

As it happens in monolinguals, the pseudohomophone effect also results in a processing advantage (naming) or disadvantage (lexical decision) in second language readers. In lexical decision tasks, cross-lingual pseudohomophones rely on phonological transference across languages (Duyck, 2005). The phonological activation of a real word in either language competes with the orthographical activation of a non-word. In the case of bilinguals, the coactivation of L1 and L2 phonologies must be handled by activating the target language and inhibiting the non-target language (Grainger and Dijkstra, 1992; van Heuven et al., 1998). Thus, pseudohomophones can have the phonology-to-orthography correspondences of the target (/dreem/ for dream) or the non-target (/drim/for dream) language of the bilingual.

To date, research about pseudohomophone interference effects in second language learners of English has focused mainly on native speakers of orthographies like Dutch or French (Nas, 1983; Duyck, 2005; Haigh and Jared, 2007; Jared et al., 2012; Commissaire et al., 2019). These authors describe pseudohomophone effects as a result of the coactivation of both languages. However, Dutch and French orthographies are not as shallow as Spanish (Seymour et al., 2003), so there is no information about how readers of more shallow orthographies behave when learning to read using a deeper orthography. The present investigation is designed to provide new insights on this topic.

Furthermore, most of the research of pseudohomophones in second language learners has been conducted in adult populations (Nas, 1983; Haigh and Jared, 2007), with a smaller number investigating teenagers (Commissaire et al., 2019) or children (Jared et al., 2012). These studies did not systematically evaluate the developmental evolution of bilingual reading acquisition. Pseudohomophone effects might not emerge in beginner readers because their orthographic representations are not formed yet. In those without orthographic representations, the conflict between phonological and orthographical information would not exist, and therefore, the incongruity that leads to a delayed response would not emerge. Changes across grades in literacy patterns have been documented in Spanish children learning English as a second language (Howard et al., 2012; Hevia-Tuero et al., 2021), and just before middle-childhood, there is a key period in which children are proficient enough to rely on lexical retrieval and they depend less on sublexical decoding (Rau et al., 2014). Nevertheless, there is no information about how this pattern may affect performance in a pseudohomophone task.

Differences between languages may lead to different reading strategies during literacy acquisition, especially when native language orthography is shallower (Spanish), and second language orthography is deeper (English). A better understanding of the factors that affect word recognition across languages with different orthographies will lead to better approaches to reading instruction in second language learners. The present investigation contributes to the literature by measuring the effect of phonological cross-linguistic interferences in Spanish children learning English (a deeper orthography language). Studies that have investigated how Spanish influences English in second language learners have focused on vocabulary, morphological awareness, reading-aloud, or spelling (Zutell and Allen, 1988; Fashola et al., 1996; Sun-Alperin and Wang, 2008; Howard et al., 2012; Goodwin et al., 2015). To our knowledge, this is the first study to investigate the effects of L1 and L2 phonology in Spanish children learning to read in English.

This research has numerous educational implications. Instructional methods influence bilingual children reading abilities (Bialystok et al., 2005b). Depending on the school’s characteristics, instructional methods expose children to different amounts of oral and written input in their different languages. For instance, reading skills in first language are important (Cummins, 1979; Maurer et al., 2021), but the amount of input received in second language also has a strong impact on reading proficiency (Matusevych et al., 2017; Mahmoud Al-Zoubi, 2018). Increased exposure to a language would mean more opportunities to process words, which may facilitate the formation of orthographic representations, as well as consolidate grapheme-to-phoneme correspondences. In Spain, there are different approaches to help children become proficient in English; however, not all of them seem to be successful (Martínez Agudo, 2019). Developing empirically validated instructional methods that are effective at teaching children to understand and read English are essential (Freeman and Freeman, 2006).

A novelty of the present study is that we measured participants responses using the computer software MouseTracker (J. Freeman and Ambady, 2010). The mouse-tracking paradigm has been extensively used in psycholinguistics research (Spivey et al., 2005; Barca and Pezzulo, 2015; Incera and McLennan, 2016; Incera, 2018). In line with previous research, the mouse-tracking paradigm measures errors and reaction times, so direct comparisons with other studies can be performed. In addition, it measures mouse trajectories (i.e., participant’s computer mouse movements as they respond to the task), which provide detailed information about the online decision-making processes taking place. Through the analysis of x-coordinates over time (how close the mouse is from the correct response) it is possible to visualize the slope of the mouse trajectory. Steeper mouse trajectories mean that responses are more efficient (the computer mouse moves faster/straighter toward the correct response). Less steep mouse trajectories mean that responses are less efficient (the computer mouse moves slower/deviates more when moving toward the correct responses).

In the present investigation, children responded to a visual lexical decision task that included English words (dream), pseudohomophones following Spanish (L1) phonological rules (drim), and pseudohomophones following English (L2) phonological rules (dreem). Children were asked to click on the green tick when reading a real word and to click on the red cross when reading a string of letters that was not an English word. Clicking on the red cross (non-word) when reading a pseudohomophone is likely to take additional time, as children would be activating the real word phonology and the incorrect orthography. Thus, using the mouse-tracking paradigm we expect responses to pseudohomophones to result in more errors, slower reaction times, and less efficient mouse trajectories for children with less English proficiency (younger children, children attending the monolingual school). We want to determine the extent to which school (monolingual, bilingual) and grade (2nd, 4th, and 6th) influence second language reading.

The present study is the first to investigate the combine effects that grade and school have on the phonological development of Spanish children learning to read in English. Grade is an important factor to consider, as reading processes quickly evolve during the elementary school years. Furthermore, instructional method is likely to have a big impact on the ability of Spanish children to read in English. While all children in Spain are required to learn English, those in schools with bilingual instructional methods are likely to be exposed to English more often than those in other schools. For each of the three types of stimuli (English Words, Pseudohomophones following Spanish phonological rules, and Pseudohomophones following English phonological rules), our predictions are:

1. Children in higher grades will perform better than children in lower grades (Main effect of Grade).
2. Children in the bilingual school will perform better than children in the monolingual school (Main effect of School).
3. The effect of school (better performance in the bilingual school) will be larger for children in higher grades (Grade by School Interaction).

## Materials and Methods

### Participants

Spanish native children from second, fourth, and sixth grade who attended two different types of schools participated in the study. All the schools that agreed to participate in the experiment were located in Spain, and they declared having a Spanish–English bilingual learning program. They were similar in terms of educational approaches during lessons taking place in Spanish. However, distinct instructional methods with respect to English were applied, and the hours per week that children were exposed to English differed. Henceforward, we will refer to them as monolingual (with less exposure to English) and bilingual (with more exposure to English) schools.

#### Monolingual School

In the monolingual school type, all the staff are Spanish native speakers. Children attend 4 h of English lessons per week and follow a Content and Language Integrated Learning methodology (CLIL; Martínez Agudo, 2019). Lessons of two other subjects, which vary depending on the grade (e.g., arts or science), also take place in English. Children are exposed to oral English during kindergarten stages through songs and letter names learning, but English instruction begins to place value on grammar and written vocabulary at Elementary levels. No specific reading instructional method is followed for English.

#### Bilingual School

The bilingual school type has some native English speakers as staff members. Lessons are taught 50% of the time in Spanish and 50% of the time in English. The instructional method emphasizes oral communication during English lessons. During kindergarten stages, children learn phonics, with explicit instruction of phonological correspondences and decoding skills. Teaching of foundation skills of reading continues in later stages, where reading and writing is combined with oral communication.

The sample included 168 participants between 7 and 12 years old (*M*_age_ = 9.60; SD_age_ = 1.60). Children were randomly recruited from both types of school, and samples were equivalent −84 from the monolingual and 84 from the bilingual school. Across both types of school, the sample included 54 children from second grade (27 males and 27 females), 58 children from fourth grade (30 males, 28 females), and 56 children from sixth grade (28 males and 28 females; see Table 1). All of them had Spanish as their native language, and they had been studying English for at least 4 years by the time of data collection. None of the participants had cognitive or behavioral impairments. Children from both types of school were socio-economically equivalent.

**Table 1:** Age and sex per school and grade.

School	Grade	Age Mean (SD)	Sex
Monolingual	Second	7.74 (0.29)	15 F/14 M
	Fourth	9.61 (0.28)	13 F/16 M
	Sixth	11.63 (0.28)	14 F/14 M
Bilingual	Second	7.67 (0.30)	12 F/15 M
	Fourth	9.61 (0.27)	15 F/14 M
	Sixth	11.63 (0.28)	14 F/14 M

### Materials

**Figure 1:**
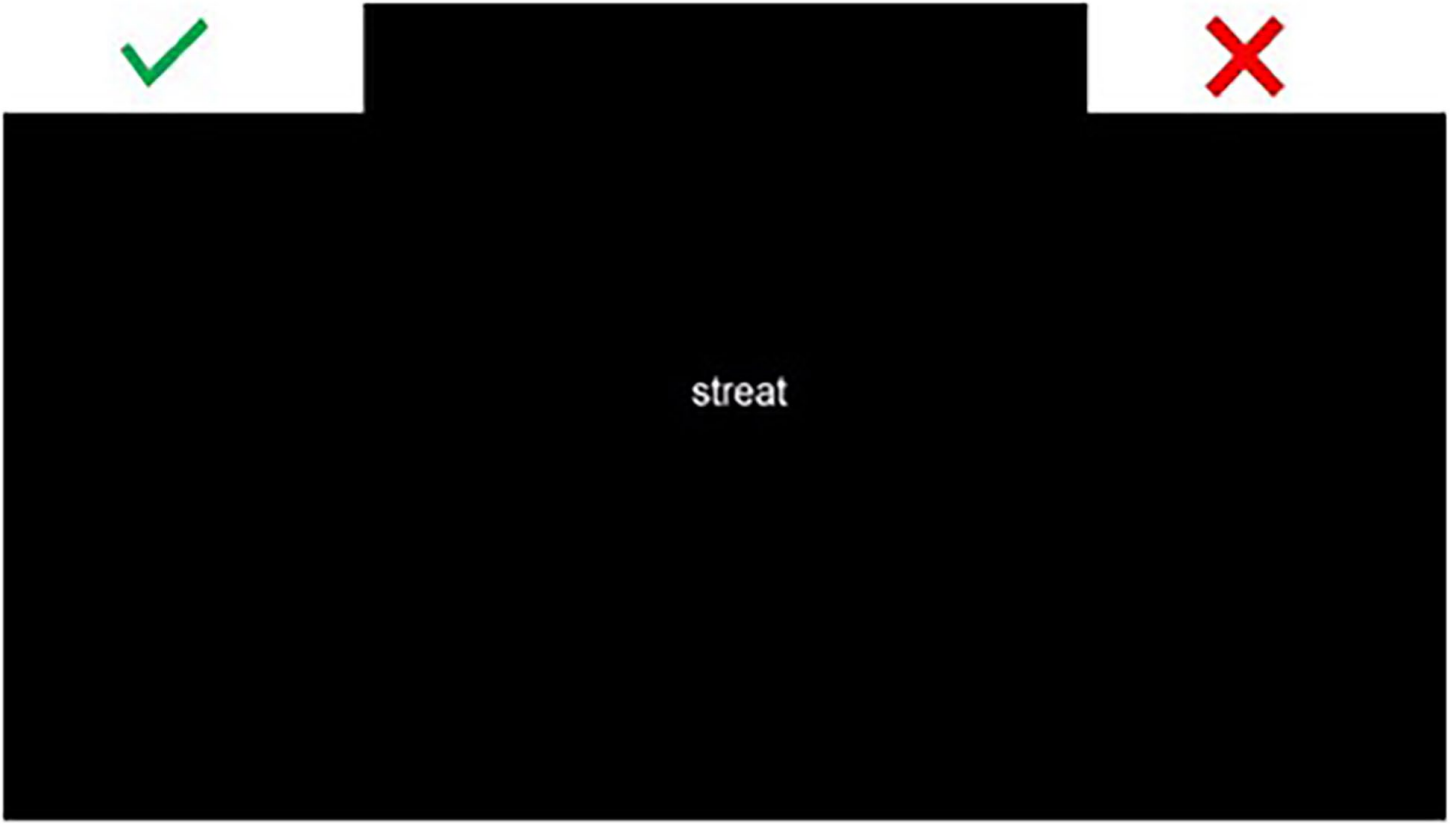
Screenshot of the participants’ view of the lexical decision task.

A total of 24 words were selected, avoiding cognates and words that could be similar in Spanish and English. The mean length was 4.54 (*SD* = 0.72) characters and the mean frequency was 55,722 according to the Subtlex-UK database (van Heuven et al., 2014). Each word (e.g., *cheese*) was manipulated in order to create a pseudohomophone with a transcription that followed Spanish phonological rules (e.g., *chis*), and a pseudohomophone with a transcription that followed English phonological rules (e.g., *chease*). Four different versions of the experiment were created in order to counterbalance the stimuli across conditions. Every participant answered to all words, but within each version of the experiment, each word appeared only in one format (word, Spanish pseudohomophone, English pseudohomophone, illegal non-word). Furthermore, stimuli were randomly presented and the position of the response options was counterbalanced. For half the participants, the “it is a word” response (green tick image) was placed on the top left corner of the screen, while for the other half the correct response was placed on the top right corner of the screen (see Figure 1). Each participant responded to 42 trials (six baseline trials, six words, six English pseudohomophones, six Spanish pseudohomophones, six illegal non-words, and 12 filler words) for a total of 7,056 observations. The illegal version of each word and other English words were included as fillers. This was necessary to balance the amount of trial types answered by each participant (same amount of real word/non-word trials).

### Procedure

The task was created with the computer software MouseTracker (Freeman and Ambady, 2010). An HP x360 Stream laptop was used to present the stimuli to the participants. Participants were asked to answer using a computer mouse and a large mouse pad (17.8 by 15.5 inches). Participants were tested individually, and performance feedback was not provided. Testing took place in a room free of noise and distracting elements to ensure the accuracy of the results. Each participant was randomly assigned to one of the eight versions of the experiment (to counterbalance the stimuli type and the response position).

Before the experiment, children were asked to complete a baseline task (Incera and McLennan, 2018; Hevia-Tuero et al., 2021). Non-linguistic trials (click on the smiley face on the top right or left corners of the screen) were included as a baseline motor task to measure the basic mouse movement abilities of the children. Furthermore, training trials were presented with the purpose of familiarizing the children with the computer program and the task before presenting the target trials.

At the beginning of each trial, START appeared at the bottom-center of the screen and the response options appeared on the top left and right corners. The written word or non-word was displayed in the center of the screen as soon as participants clicked START. The stimuli remained on the screen until participants clicked on one of the two response alternatives (green tick for real words, red cross for non-words). Children were told to click on one of the two response options as quickly and accurately as possible. Once they answered, the START button appeared and they had to click on it to initiate the next trial. If participants took more than 750 milliseconds to initiate a mouse movement, a warning appeared instructing them to start moving the mouse earlier on in future trials.

### Analysis Plan

R-software (version 4.0.2) was used to run the mixed model analyses using the lme4 package (version 1.1–21; Bates et al., 2015). To analyze number of errors, we combined the advantages of ordinary logit models with the ability to account for random subject and item effects (Jaeger, 2008). The independent variables included in the analyses were grade (2nd, 4th, and 6th) and school (monolingual, bilingual). We performed separate analyses for each of the three types of stimuli in the lexical decision task: Words, Pseudohomophones following Spanish phonological rules, and Pseudohomophones following English phonological rules. The dependent variables included in the analyses were number of errors, reaction times, and mouse trajectory (x-coordinates over time).

The MouseTracker program measures participants’ mouse positions over time, which includes three variables: *y*-coordinates, *x*-coordinates, and time (in milliseconds). Since three-dimensional graphs are hard to visualize, the standard in the field is to report *x*-coordinates over time [see (Incera, 2018), for a detailed discussion of methodological concerns and practical recommendations when using the mouse-tracking paradigm with bilingual populations]. While all participants move the mouse upwards (START is at the bottom and the response options are at the top of the screen) the way in which the task is set up results in the manipulation influencing whether participants move right or left (toward the response options on the right or left corner). Thus, we report mouse trajectories as *x*-coordinates over time.

Outliers were filtered, deleting correct responses with reaction times over and under 2 SD for each school, grade, and type of stimuli. First, we performed the Grade by School analysis on the baseline, in order to determine whether children in both schools are equivalent at the motor level. The baseline analysis does not include the random effect of items because all trials are the same (at baseline there is no item variability to account for). Second, we performed the Grade by School analysis on words, pseudohomophones following Spanish rules, and pseudohomophones following English rules. The goal was to test the effect of Grade (children in higher grades perform better), the effect of School (children in the bilingual school perform better), and the Grade by School interaction (the effect of school—bilingual better—is larger in higher grades). Random effects of participants and items were included crossed in all models testing Words, Spanish Pseudohomophones, and English Pseudohomophones. Models were compared using the Chi-square test; only factors that significantly contributed to model fit, as determined by a significant value of *p* in the chi-square test, were included in the final model. The estimate (effect size) and standard error of each effect was reported for all factors included in the final model for each dependent variable.

## Results

The data and the R Notebook with the analyses can be found at the Open Science Framework.

### Errors

Errors are calculated by counting the number of times children clicked on the incorrect response (red cross for words, green tick for pseudohomophones). Error analyses cannot be conducted for the baseline task since there are no errors; all children were able to click on the smiley face at the top right/left corner of the screen without making any mistakes.

**Figure 2:**
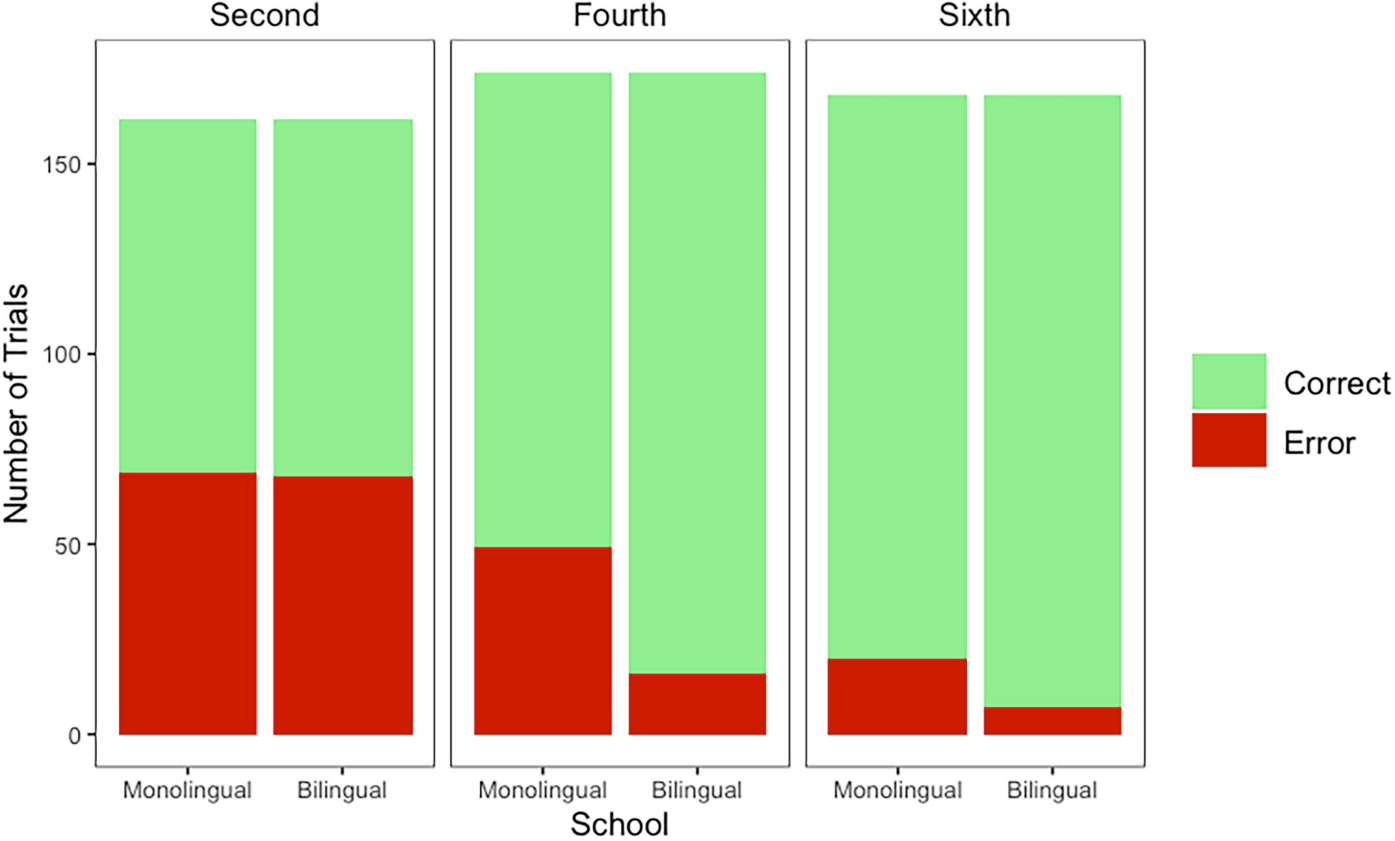
Correct answers (green) and errors (red) per grade and school for words.

When analyzing number of errors for *words*, model comparisons indicated that there was a main effect of Grade [*χ*^2^_(2)_ = 80.44, *p* < 0.001] and a main effect of School [*χ*^2^_(1)_ = 13.32, *p* < 0.001], in line with our first and second hypotheses. Furthermore, the Grade by School interaction [*χ*^2^_(2)_ = 11.56, *p* = 0.003] also improved model fit, in line with our third hypothesis. The final model for errors for words as modeled in R is as follows: Error ~ Grade*School + (1|Participant) + (1|Stimuli). The effect of Grade emerged because second graders had more errors than fourth (*Estimate* = −0.84, *SE* = 0.33) and sixth (*Estimate* = −2.24, *SE* = 0.39) graders. The interaction emerged because, while the number of errors in words was equivalent for monolingual and bilingual children in second grade (second grade monolinguals 42.59%; second grade bilinguals 41.97%), the monolingual children had more errors than the bilingual children in fourth (fourth grade monolinguals 28.16%; fourth grade bilinguals 9.19%; *Estimate* = −1.70, *SE* = 0.52) and sixth (sixth grade monolinguals 11.90%; sixth grade bilinguals 4.16%; *Estimate* = −1.28, *SE* = 0.62) grades (Figure 2).

**Figure 3:**
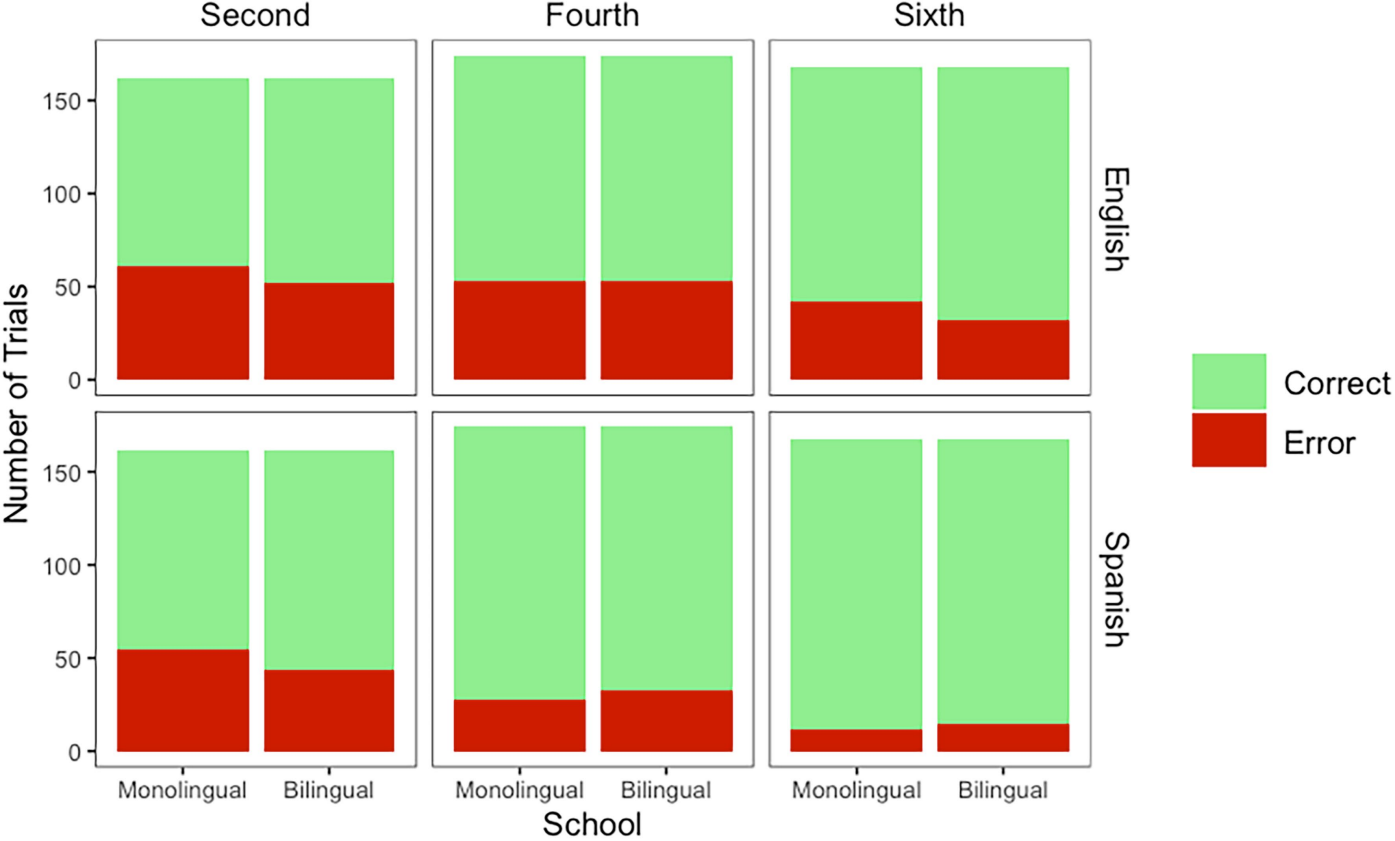
Correct answers (green) and errors (red) per grade and school for Spanish and English pseudohomophones.

In the *Spanish pseudohomophones* analysis, model comparisons indicated that there was a main effect of Grade [*χ*^2^_(2)_ = 40.41, *p* < 0.001]. However, there was no effect of School [*χ*^2^_(1)_ = 0.04, *p* = 0.841] and there was no Grade by School interaction [*χ*^2^_(2)_ = 2.38, *p* = 0.303]. The final model for errors for Spanish pseudohomophones as modeled in R is as follows: Error ~ Grade + (1|Participant) + (1|Stimuli). The effect of Grade emerged because children in second grade had more errors than children in fourth (*Estimate* = −0.90, *SE* = 0.26) and sixth (*Estimate* = −1.92, *SE* = 0.31) grades. In the *English pseudohomophones* analysis, model comparisons indicated that there was a main effect of Grade [*χ*^2^_(2)_ = 11.13, *p* = 0.003]. However, the effect of School [*χ*^2^_(1)_ = 1.58, *p* = 0.208] and the Grade by School interaction [*χ*^2^_(2)_ = 0.917, *p* = 0.631] did not emerge. The final model for errors for English pseudohomophones as modeled in R is as follows: Error ~ Grade + (1|Participant) + (1|Stimuli). While there were no differences between children in second and fourth grade (*Estimate* = −0.24, *SE* = 0.22), the effect of Grade emerged because there were differences between children in second and sixth grade (*Estimate* = −0.74, *SE* = 0.22), the older children had less errors (see Figure 3).

In sum, error analyses for Words supported Hypothesis 1 (Effect of Grade), Hypothesis 2 (Effect of School), and Hypothesis 3 (Grade by School Interaction). Furthermore, error analyses for Pseudohomophones supported Hypothesis 1 (Effect of Grade). However, the effect of School (Hypothesis 2) and the Grade by School Interaction (Hypothesis 3) did not emerge in error analyses for pseudohomophones. Children from both schools (monolingual, bilingual) were equally likely to consider the pseudohomophones incorrect.

### Reaction Times

Reaction times were measured from the moment the stimulus appeared on the screen to the moment participants clicked on the response. When analyzing the *baseline*, model comparisons indicated that there was a main effect of Grade [*χ*^2^_(2)_ = 73.01, *p* < 0.001] and a main effect of School [*χ*^2^_(1)_ = 4.61, *p* = 0.031]. The Grade by School interaction did not emerge [*χ*^2^_(2)_ = 1.49, *p* = 0.472]. The final model for reaction times for baseline as modeled in R is as follows: RT ~ Grade + School + (1|Participant). The effect of Grade emerged because second graders responded 499 ms (*SE* = 83) slower than fourth graders, and 808 ms (*SE* = 84) slower than sixth graders. The effect of School emerged because children from the bilingual school responded 147 ms (*SE* = 68) faster than children from monolingual school (see Table 2).

**Table 2:** Descriptive statistics (means and standard deviations) for reaction times responding to each condition (English Words, Spanish Pseudohomophones, English Pseudohomophones) per grade and school.

School	Grade	Baseline	Word	Spanish	English
Monolingual	Second	2,177 (886)	3,161 (1147)	3,441 (1203)	3,760 (1350)
	Fourth	1,579 (652)	2,484 (962)	2,619 (884)	2,698 (919)
	Sixth	1,293 (406)	1,942 (528)	2,133 (609)	2,293 (705)
Bilingual	Second	1,912 (679)	2,750 (1111)	3,256 (1303)	3,559 (1376)
	Fourth	1,510 (1016)	1,977 (546)	2,497 (881)	2,438 (718)
	Sixth	1,179 (450)	1,710 (433)	1,870 (452)	2,019 (519)

When analyzing *words*, model comparisons indicated that there was a main effect of Grade [*χ*^2^_(2)_ = 86.60, *p* < 0.001] and a main effect of School [*χ*^2^_(1)_ = 17.61, *p* < 0.001]. However, the Grade by School interaction did not emerge [*χ*^2^_(2)_ = 1.91, *p* = 0.385]. The final model for reaction times for words as modeled in R is as follows: RT ~ Grade + School + (1|Participant) + (1|Stimuli). The effect of Grade emerged because second graders responded 782 ms (*SE* = 109) slower than fourth graders, and 1,208 ms (*SE* = 108) slower than sixth graders. The effect of School emerged because children from bilingual school responded 374 ms (*SE* = 86) faster than children from monolingual school (see Table 2).

When analyzing *Spanish pseudohomophones*, there was a main effect of Grade [*χ*^2^_(2)_ = 88.03, *p* < 0.001]. The main effect of School [*χ*^2^_(1)_ = 3.78, *p* = 0.051] and the Grade by School interaction did not emerge [*χ*^2^_(2)_ = 0.28, *p* = 0.868]. The final model for reaction times for Spanish pseudohomophones as modeled in R is as follows: RT ~ Grade + (1|Participant) + (1|Stimuli). Overall, children took more than 3,000 ms to respond (*Estimate* = 3,400, *SE* = 96). Second graders were 780 ms slower than fourth graders (*SE* = 129) and 1,389 ms slower than sixth graders (*SE* = 129). When analyzing *English pseudohomophones*, there was a main effect of Grade [*χ*^2^_(2)_ = 105.85, *p* < 0.001] and a main effect of School [*χ*^2^_(1)_ = 7.46, *p* = 0.006]. The Grade by School interaction [*χ*^2^_(2)_ = 0.03, *p* = 0.984] did not emerge. The final model for reaction times for English pseudohomophones as modeled in R is as follows: RT ~ Grade + School + (1|Participant) + (1|Stimuli). Overall, children took more than 3,500 ms to respond (*Estimate* = 3,869, *SE* = 111). Second graders were 1,111 ms (*SE* = 129) slower than fourth graders and 1,555 ms (*SE* = 128) slower than sixth graders. Children attending a bilingual school were 286 ms (*SE* = 103) faster than children attending a monolingual school (see Table 2).

In sum, reaction time analyses for Words supported Hypothesis 1 (Effect of Grade) and Hypothesis 2 (Effect of School), but not Hypothesis 3 (Grade by School Interaction). Furthermore, reaction time analyses for Pseudohomophones supported Hypothesis 1 (Effect of Grade). Interestingly, the reaction time effect of School (Hypothesis 2) emerged in English but not in Spanish Pseudohomophones. Finally, the Grade by School Interaction (Hypothesis 3) did not emerge for Pseudohomophones.

### Mouse Trajectories

Mouse trajectories are measured with *x*-coordinates over time. When analyzing the *baseline*, model comparisons indicated that on the slope of the mouse trajectory there was a main effect of Grade [*χ*^2^_(2)_ = 48.10, *p* < 0.001]. However, the main effect of School [*χ*^2^_(1)_ = 1.53, *p* = 0.215] and the Grade by School [*χ*^2^_(5)_ = 2.26, *p* = 0.811] interaction did not emerge. The final model for mouse trajectories for baseline as modeled in R is as follows: X100 ~ Time*Grade + (Time|Participant). The effect of Grade emerged on the slope of the mouse trajectories (Time*Grade) because, when compared to children in second grade, the mouse trajectories were steeper (better performance) for children in fourth (*Estimate* = −2.14, *SE* = 0.68) and sixth grade (*Estimate* = −5.09, *SE* = 0.68).

**Figure 4:**
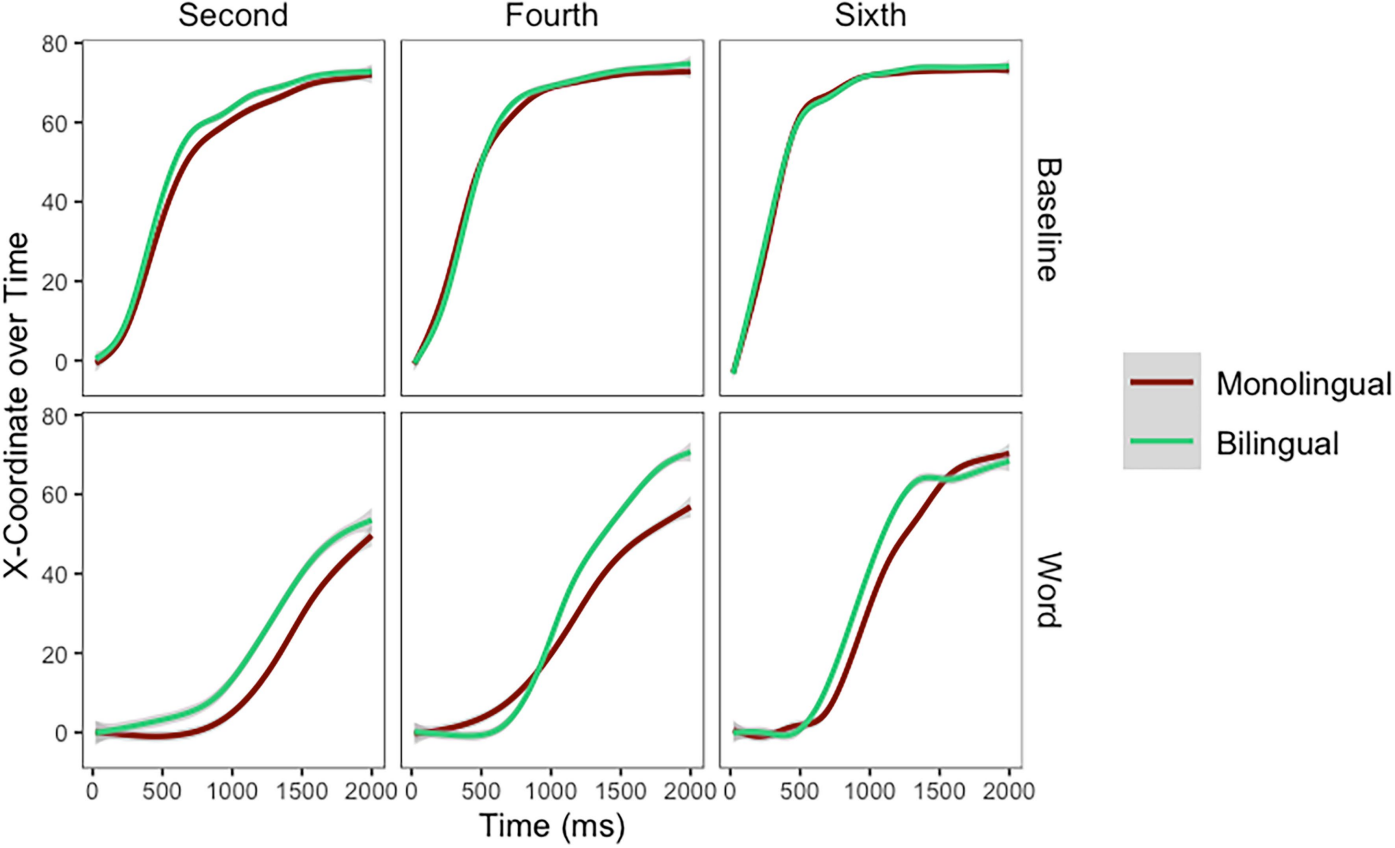
Mouse trajectories for baseline and words per grade and school.

In *words*, model comparisons indicated that on the slope of the mouse trajectory there was a main effect of Grade [*χ*^2^_(2)_ = 38.97, *p* < 0.001] and a Grade by School [*χ*^2^_(5)_ = 15.03, *p* = 0.010] interaction. The main effect of School [*χ*^2^_(1)_ = 1.43, *p* = 0.231] did not emerge. The final model for mouse trajectories for words as modeled in R is as follows: X100 ~ Time*Grade*School + (Time|Participant) + (1|Stimuli). The effect of Grade emerged on the slope of the mouse trajectories (Time*Grade) because—compared to children in second grade—the mouse trajectories were steeper (better performance) for children in fourth (*Estimate* = 4.24, *SE* = 2.28) and sixth grade (*Estimate* = 12.30, *SE* = 2.27). The Grade by School interaction emerged on the slope of the mouse trajectories (Time*Grade*School) because the difference between the children attending the monolingual and the bilingual school was larger in fourth than second grade (*Estimate* = 4.56, *SE* = 3.19). However, the difference was smaller in sixth than second grade (*Estimate* = −2.74, *SE* = 3.20). While in sixth grade the children attending the bilingual school still outperformed the children attending the monolingual school (see Figure 4), this difference—the effect of school—was not as large in sixth as in fourth grade.

**Figure 5:**
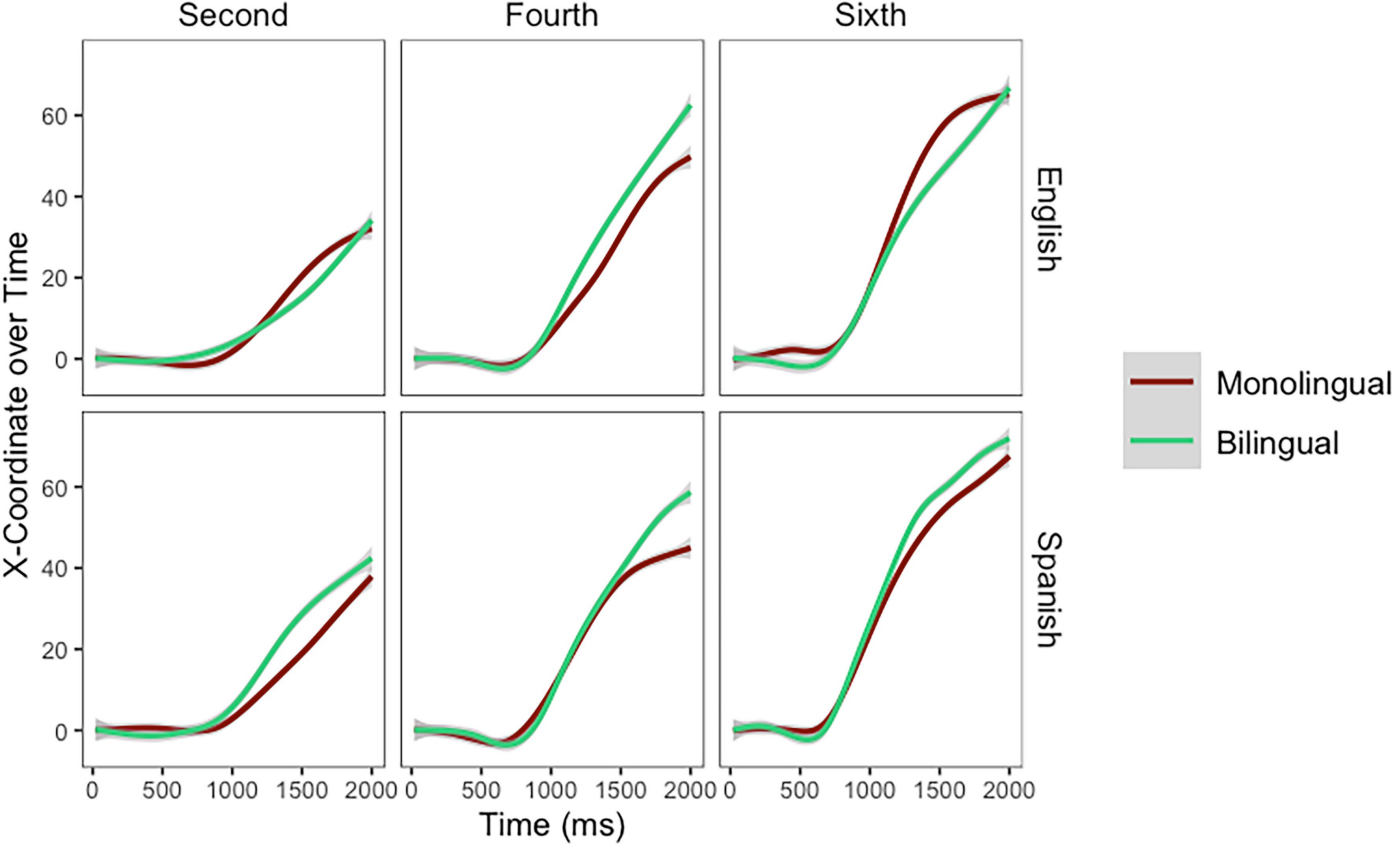
Mouse trajectories for Spanish and English pseudohomophones per grade and school.

In *Spanish pseudohomophones*, model comparisons indicated that on the slope of the mouse trajectory there was a main effect of Grade [*χ*^2^_(2)_ = 47.85, *p* < 0.001]. However, the main effect of School [*χ*^2^_(1)_ = 0, *p* = 0.994] and the Grade by School [*χ*^2^_(5)_ = 6.70, *p* = 0.243] interaction did not emerge. The final model for mouse trajectories for Spanish pseudohomophones as modeled in R is as follows: X100 ~ Time*Grade + (Time|Participant) + (1|Stimuli). When responding to Spanish pseudohomophones, mouse trajectories were steeper for children in fourth (*Estimate* = 5.39, *SE* = 1.78) and sixth (*Estimate* = 13.23, *SE* = 1.79) grades. In *English pseudohomophones*, there was a main effect of Grade [*χ*^2^_(2)_ = 46.27, *p* < 0.001]. However, the main effect of School [*χ*^2^_(1)_ = 0.02, *p* = 0.864] or the Grade by School [*χ*^2^_(5)_ = 3.18, *p* = 0.67] interaction did not emerge. The final model for mouse trajectories for English pseudohomophones as modeled in R is as follows: X100 ~ Time*Grade + (Time|Participant) + (1|Stimuli). When responding to English pseudohomophones, mouse trajectories were steeper for children in fourth (*Estimate* = 9.27, *SE* = 1.94) and sixth (*Estimate* = 14.12, *SE* = 1.96) grades (see Figure 5).

In sum, all analyses performed on the Mouse Trajectories supported Hypothesis 1 (Effect of Grade). However, Hypothesis 2 (Effect of School) did not emerge for words or pseudohomophones. Finally, results from Words support Hypothesis 3 (Grade by School interaction), but fourth grade (as opposed to sixth) is where the effect of School is largest.

### Exploratory Analysis

**Figure 6:**
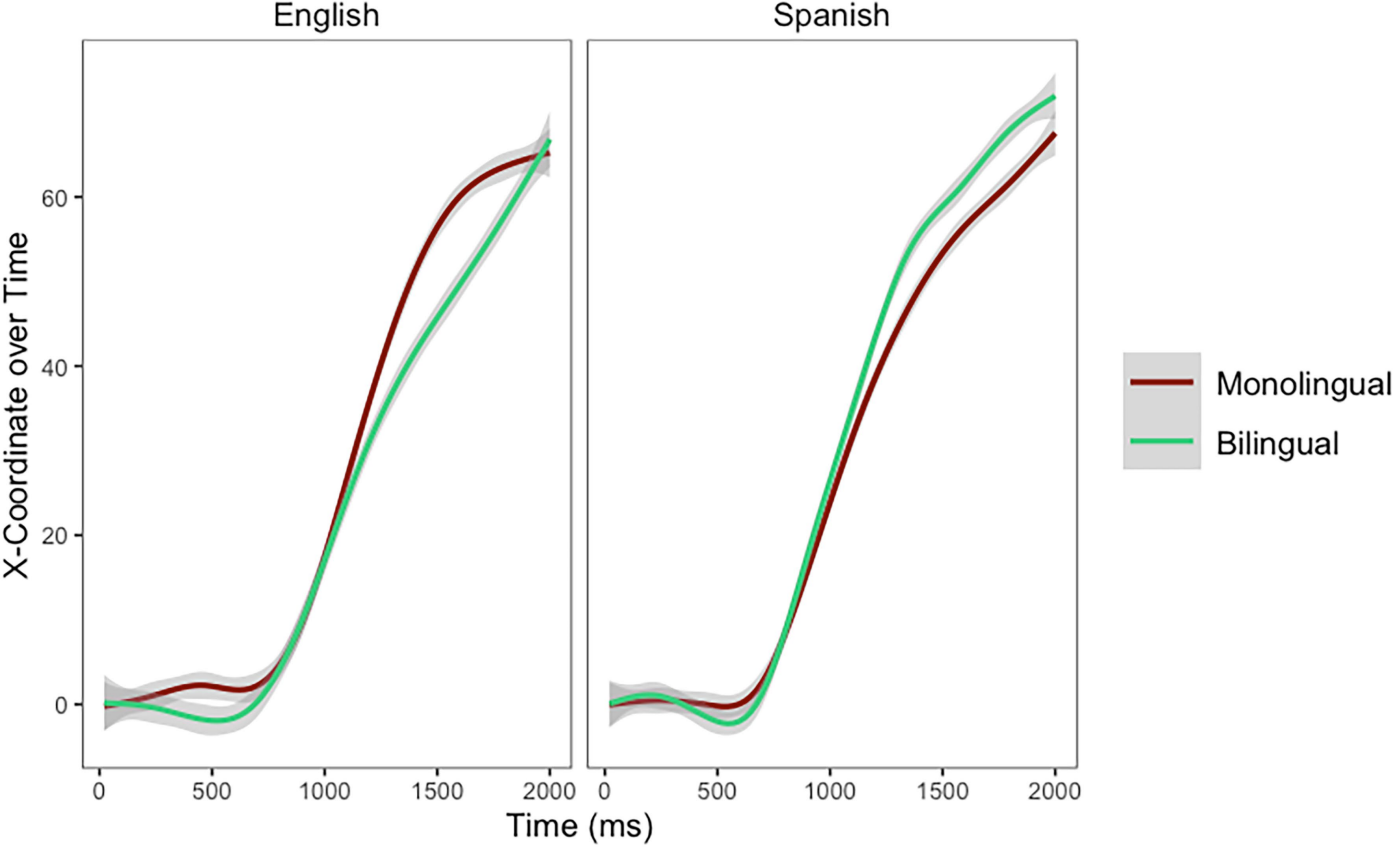
Mouse trajectories for Spanish and English pseudohomophones per school in sixth grade.

The intriguing pattern of results for sixth graders in Figure 5 (the children from the monolingual school seem to outperform the children from the bilingual school) led to an *exploratory analysis* performed on the slope of the mouse trajectories. This analysis focuses exclusively on children in sixth grade (the group where this interaction seems to emerge). The goal is to explore the potential Pseudohomophone (Spanish, English) by School (Monolingual, Bilingual) interaction in these skilled children. When sixth graders responded to the pseudohomophones, there was a main effect of Pseudohomophone [*χ*^2^_(1)_ = 67.36, *p* < 0.001] and a Pseudohomophone by School interaction [*χ*^2^_(3)_ = 215.87, *p* < 0.001]. The final model for this exploratory analysis as modeled in R is as follows: X100 ~ Time*Condition*School + (Time|Participant) + (1|Stimuli). The main effect of Pseudohomophone emerged because for all students English pseudohomophones were more distracting than Spanish pseudohomophones (*Estimate* = 0.28, *SE* = 0.39). However, the main effect of School [*χ*^2^_(1)_ = 0.06, *p* = 0.805] did not emerge. The cross-over interaction (see Figure 6) emerged because children from the bilingual school outperformed children in the monolingual school when responding to Spanish Pseudohomophones, while children in the monolingual school outperformed children in the bilingual school when responding to English pseudohomophones (*Estimate* = 4.99, *SE* = 0.55).

Close inspection of Figure 6 indicates that early in the trajectory (around 500 ms after stimulus onset) children in the bilingual school are more distracted by both types of pseudohomophones than children in the monolingual school. Once participants start moving toward the correct response, the interaction emerges. Children in the bilingual school are able to outperform their counterparts in Spanish pseudohomophones, but children in the monolingual school outperform their counterparts in English pseudohomophones.

## Discussion

The aim of this study is to determine how native language (i.e., Spanish) interferes with second language reading (i.e., English); especially when L1 is shallower (phonemes and graphemes are more consistently linked) than L2. Importantly, we explored the extent to which grade (2nd, 4th, and 6th) and type of school (Bilingual, Monolingual) play a role in the acquisition of L2 grapheme-to-phoneme correspondence rules.

### Words

In line with Hypothesis 1, older children performed better than younger children. Sixth graders had less errors, faster reaction times, and straighter mouse trajectories than fourth and second graders. In line with Hypothesis 2, children attending a bilingual school performed better (less errors, faster reaction times) than children attending a monolingual school. In line with Hypothesis 3, the effect of school (Spanish children attending a bilingual school having less errors) was larger in higher grades. In second grade, children attending the bilingual and the monolingual schools had similar number of errors (close to 40%) when recognizing English words. While students in the second grade performed above chance (60% accuracy), performance substantially improved in higher grades, especially for the children attending the bilingual school.

Our results from number of errors in words point to the conclusion that early on children in both schools perform equally, but as time passes children in the bilingual school outperform those in the monolingual school. Considering the MROM-p (Jacobs et al., 1998), word nodes are strongly activated in children attending a bilingual school, facilitating their responses in the lexical decision task and increasing processing speed in visual word recognition. The fact that the differences between bilinguals and monolinguals are larger in older children indicates that the effect of type of school is cumulative. Those in the bilingual school continue gaining advantages until at least sixth grade, as they are likely to have a higher level of English exposure (in particular, oral exposure).

Regarding the development of the orthography, higher exposure to English among bilingual children could have benefited their formation of strong orthographic representations along their schooling experience. These strong representations could have made their visual word recognition more accurate. An increase of English instructional time, especially the increase in oral instructional time, could have aided the bilingual children in consolidating the grapheme-to-phoneme correspondences. In addition, more opportunities to form orthographic representations are gained with more exposure to written words, which allows for a more efficient transition from serial phonological decoding to lexical processing (Share, 1995). This would be especially relevant for English reading acquisition due to the opacity of the English orthography. In English, phonological decoding is not enough to process words (Cunningham et al., 2002; Ziegler and Goswami, 2005). Finally, having more vocabulary is likely to facilitate recognition of a higher number of words, which makes readers more confident at rejecting non-words. This last possibility is supported by our results as children attending the monolingual school were less confident at rejecting non-words. The number of errors suggests that real English words were not recognized as such by the children in the monolingual school, likely because these children do not know these words yet.

Reaction times indicated that children from the bilingual school were faster than children from the monolingual school. A caveat to claim an advantage is that children in the bilingual school were also faster at baseline. In order to conclude that there are cognitive effects at play, the effect needs to be above and beyond that of the baseline. Indeed, the time difference between children attending the monolingual and bilingual school when responding to English words (374 ms) was more than double that the difference between these two groups at baseline (147 ms). Even though this effect needs to be considered cautiously—the two groups were not equivalent at baseline so the difference could be due (at least in part) to motor influences—the results indicate that the bilingual school has a positive effect on reading performance.

While baseline differences emerged in reaction times they did not emerge in mouse trajectories. Mouse trajectories showed that children were equivalent in terms of baseline mouse movements. When looking at the effect of school on the mouse trajectory, those attending the bilingual school were better at processing English words than those attending the monolingual school. Interestingly, this effect was largest in fourth grade. This is an important finding as it points to a time in development when the effect of School might be maximal (at least when measuring performance using a Lexical Decision task). Alternatively, it is possible that the task was too easy for the older children, thus the difference does not emerge because the sixth graders are performing at ceiling.

### Pseudohomophones

In line with Hypothesis 1, when responding to pseudohomophones older children performed better than younger children. Second graders were more affected by the pseudohomophones than older students, probably because they do not have strong English orthographic representations. Second graders had recently started English literacy learning, and correspondence rules might not have been well established at this stage. Additionally, less expertise in L1 inhibition, joined to a lack of reading proficiency, are likely to result in less efficient reading performance. As Hamada (2017) observed, the influence of the native language phonology decreases when learners become more proficient in second language. A difference in knowledge of English phonological rules between children in the bilingual and the monolingual school could also explain these effects. There were no differences between schools with respect to errors in pseudohomophones. The nature of the cognitive processes at play (rejecting a non-word vs. accepting a word) is likely to have influenced these results. All children, even those in the bilingual school, took time and had doubts when rejecting the pseudowords and accidentally accepted some pseudowords as real words. Further research is necessary to determine what additional variables (e.g., oral versus written exposure) are influencing pseudohomophone effects in bilinguals.

While the main effect of school—children in the bilingual school outperforming children in the monolingual school—did not emerge in mouse trajectories, we observed a cross-over interaction. In line with Hypothesis 3, when responding to Spanish pseudohomophones children in the bilingual school outperformed children in the monolingual school. However, against Hypothesis 3, when responding to English pseudohomophones, children in the monolingual school outperformed their peers attending the bilingual school. In sixth grade, children attending a bilingual school are very efficient at rejecting Spanish pseudohomophones, but they get more distracted by the English pseudohomophones than children attending a monolingual school (Figure 6). The fact that children in the bilingual school are more confident discarding non-words that clearly follow Spanish rules than children in the monolingual school, support the idea that children in the bilingual school have more experience/practice inhibiting Spanish. The fact that children in the monolingual school get less distracted by the English pseudohomophones than children in the bilingual school indicate that their English phonology might not be as strongly developed.

### Implications

Children learning a second language automatically activate L2-specific rules during word reading. All participants had more errors when responding to English than Spanish pseudohomophones (see Figure 3). When responding to an English task (in English mode), native speakers of Spanish were more distracted by the English than the Spanish phonology. This pseudohomophone effect is equivalent in other languages (Nas, 1983; Commissaire et al., 2019). In line with other studies, Spanish children develop knowledge of English phonology relatively early during development (Hevia-Tuero et al., 2021). In our study, the pseudohomophone effect emerged even in second grade, and not only in advanced L2 learners like Commissaire and colleagues had previously reported (2019). An emerging knowledge of English orthography is acquired at early stages, with relatively few years of instruction. These results support the idea that phonological information is activated in visual word recognition (Goswami et al., 2001; Ziegler et al., 2001).

The type of school children attend to (bilingual, monolingual) influences word processing. This effect could be due to higher levels of exposure to the second language or to a different approach to reading instruction. Different instructional methods might lead to different ways of processing, altering the orthography–phonology relationship. Indeed, instructional methods and native language characteristics influence reading strategies in both native and second language (Bhide, 2015). Furthermore, phonics instruction facilitates successful learning of relationships between letters and sounds, a requirement for learning to read (Castles et al., 2018). Not having been explicitly taught about English phonics, children in monolingual schools could be building orthographic representations without developing English phonological representations. These children could be relying on the lexical route or on Spanish phonological representations. In this way, they could be processing a whole word unit and rejecting a non-word based on orthographical characteristics. Results from the monolingual school coincide with what Pitts and Hanley (2010) found in their study: Spanish-speaking adults were less reliant on phonology than native speakers, despite knowing well the English grapheme-to-phoneme rules. These findings support the triangle model of cooperation between phonology and orthography to read words (Seidenberg and McClelland, 1989; Harm and Seidenberg, 2004). For those with less knowledge of phonology, a development of a direct orthography-to-semantics pathway would be reasonable (and advantageous in this task). Children attending a bilingual school have a foundation of phonic knowledge, and they are more familiar with English phonology. Therefore, they are likely to have a balanced division of labor. This approach is efficient in some situations (when phonology is helpful). However, the activation of the English phonology makes these bilingual children more sensitive to pseudohomophone effects. The type of school children attend influence their processing strategies during word recognition. There is a shift in the division of labor between the orthographic and the phonological component, which is likely to be influenced by how much written and oral exposure they have in their second language.

The more plausible explanation for our results is that English phonology plays a major role in the way that children in bilingual schools learn. When processing English pseudohomophones, the conflict between the existence of phonological information and the lack of orthographical information of a real word makes them move toward the correct response (rejecting the pseudohomophone) less efficiently. Although they have developed a better “rejection of Spanish” mechanism than children in the monolingual schools, they are still more distracted by the English phonology. These results connect to an increase on the activation of the English language node as described in BIA model (van Heuven et al., 1998) in children attending a bilingual school. Being aware of the stimuli language membership activates the language node. Moreover, for the children attending a bilingual school the higher level of exposure to English is likely to intensify language node activation.

The performance differences found between these instructional methods are remarkable. These results open the possibility for new research in L2 literacy instruction. The goal would be to better understand how instructional methods influence reading proficiency in each language, as authors like Rolla San Francisco et al. (2006) have suggested. Attending a bilingual school may strengthen English phonology activation. While this might constitute a disadvantage in a lexical decision task involving pseudohomophones, this is likely to be helpful when reading. This way of processing written text is closer to the “native” way of processing English words, which speaks to the good job bilingual schools are doing.

The current study is one of the few studies that have investigated L1 and L2 phonology interferences in Spanish children learning English. Moreover, this is the first study to use a pseudohomophone lexical decision task for this purpose. These findings support and complement previous research about phonological activation in second language learners during reading tasks. Furthermore, the present experiment adds to the literature on pseudohomophone effects in orthographic systems with different orthographic depths (shallower, like Spanish and Dutch; or deeper, like French and English). Our results are in line with those reported by Commissaire et al. (2019) and Nas (1983). Nas (1983) focused on adult Dutch L2 learners who had reach a proficient level of reading in their native language. Commissaire et al. (2019) studied adolescent French L2 learners of sixth and eighth grades. The novelty of our study is that participants started English instruction at an early age, and they learnt to read in both languages (L1 and L2) at the same time. In fact, the ages of the children participating in this study match the age for literacy foundation, which is another important contribution of the present investigation. Evaluating children across different grades allowed us to investigate the evolution of L1 and L2 during simultaneous reading learning, shedding light on the processes of literacy acquisition of English learners. However, we do not know to what extent our findings can be extrapolated to other populations of English learners, like Chinese or Hebrew speakers. Spanish and English share the same alphabet, which may have facilitated orthographic rule learning (Pasquarella et al., 2015). Future studies should address this issue, as cross-linguistic transfer is likely to be influenced by the proximity of L1 and L2 orthographies (Geva and Siegel, 2000; Chung et al., 2019).

The mouse-tracking paradigm allowed us to explore children’s responses as they unfold over time. This methodology could be used in future studies to investigate automatic phonological activation during reading in tasks like visual masked priming using pseudohomophones (see Duyck, 2005; Ziegler et al., 2013; Sauval et al., 2017). Additionally, it would be interesting to focus on the effect of linguistic variables in order to broaden our knowledge of visual word recognition in L2 learners. Data focused on Spanish speakers learning English are scarce, despite the fact that English and Spanish are the first and fourth most commonly spoken languages in the world (Eberhard et al., 2020). Further investigations are needed to explore how reading mechanisms from the native language interfere with how children learn to read in their second language.

There are additional variables that could be taken into account when investigating these effects. Teachers were asked to select children with average reading skills, and children with difficulties were not included. This study did not assess Spanish and English reading skills, nor did it take into account domain-general abilities like inhibitory control (Bartolotti et al., 2011), which likely influence children’s performance. The practical concerns of creating a study short enough for young children, while assessing a wide range of linguistic and cognitive skills, is a real challenge. Furthermore, data were collected during school hours, so students could not be absent from class too long. Additional variables related to the school are likely to influence children’s performance. Some examples are the amount of time (only at school, also outside of school) and the type of exposure (oral versus written) to the language, the presence or absence of native speaker teachers, and the instructional methods used during pre-literacy stages. Together, these are factors that may be relevant for Spanish children learning English. It would be interesting to assess the specific weight of these variables in future studies, building on previous research (De Wilde et al., 2020).

## Conclusion

In conclusion, the aim of this experiment was to understand how Spanish children learn to read in English. We found that Spanish children are able to recognize English orthography independently of their grade and the type of school they attend (monolingual, bilingual). Interestingly, differences in teaching methodologies—like an oral emphasis in bilingual schools versus a written emphasis in monolingual schools, as well as explicit phonics instruction—influence how L2 learners read. Spanish children in the bilingual school are more efficient at recognizing English words and discarding Spanish pseudohomophones, but get more distracted by English pseudohomophones. These results are in line with the idea that children in the bilingual school have better oral English (better English phonological representations) which makes them perform similar to the way in which native English speakers perform. The way in which learners are exposed to a second language determines how they process the orthography and phonology of their languages. Instructional methods influence the strength of the L1 and L2 inhibition processes.

## Data Availability Statement

The original contributions presented in the study are included in the article/supplementary materials, further inquiries can be directed to the corresponding author.

## Ethics Statement

The studies involving human participants were reviewed and approved by Comité de Ética de la Investigación del Principado de Asturias. Written informed consent to participate in this study was provided by the participants’ legal guardian/next of kin.

## Author Contributions

CH-T made contributions to the collection, analysis, interpretation of data for the work, and drafted the manuscript. SI and PS-C supervised the study and revised it critically for important intellectual content. All authors contributed to the article and approved the submitted version.

## Funding

This study was supported by the Ministry of Science and Innovation of Spanish Government (predoctoral grant number FPU18/03368 and grant number PID2019-106868GB-I00).

## Conflict of Interest

The authors declare that the research was conducted in the absence of any commercial or financial relationships that could be construed as a potential conflict of interest.

## Publisher’s Note

All claims expressed in this article are solely those of the authors and do not necessarily represent those of their affiliated organizations, or those of the publisher, the editors and the reviewers. Any product that may be evaluated in this article, or claim that may be made by its manufacturer, is not guaranteed or endorsed by the publisher.
